# SERS substrates formed by gold nanorods deposited on colloidal silica films

**DOI:** 10.1186/1556-276X-8-250

**Published:** 2013-05-22

**Authors:** Mikhail Yu Tsvetkov, Boris N Khlebtsov, Vitaly A Khanadeev, Victor N Bagratashvili, Peter S Timashev, Mikhail I Samoylovich, Nikolai G Khlebtsov

**Affiliations:** 1 Institute of Laser and Information Technologies, Russian Academy of Sciences, 2 Pionerskaya Ulitsa, Moscow, Troitsk 142190, Russia; 2 Institute of Biochemistry and Physiology of Plants and Microorganisms, Russian Academy of Sciences, 13 Prospekt Entuziastov, Saratov 410049, Russia; 3 Central Research Technological Institute “TECHNOMASH”, 4, I. Franko Ulitsa, Moscow 121108, Russia; 4 Saratov State University, 83 Astrakhanskaya Ulitsa, Saratov 410012, Russia

**Keywords:** SERS, Gold nanorod, Silica nanosphere, Plasmonic nanopowder, Rhodamine 6G

## Abstract

We describe a new approach to the fabrication of surface-enhanced Raman scattering (SERS) substrates using gold nanorod (GNR) nanopowders to prepare concentrated GNR sols, followed by their deposition on an opal-like photonic crystal (OPC) film formed on a silicon wafer. For comparative experiments, we also prepared GNR assemblies on plain silicon wafers. GNR-OPC substrates combine the increased specific surface, owing to the multilayer silicon nanosphere structure, and various spatial GNR configurations, including those with possible plasmonic hot spots. We demonstrate here the existence of the optimal OPC thickness and GNR deposition density for the maximal SERS effect. All other things being equal, the analytical integral SERS enhancement of the GNR-OPC substrates is higher than that of the thick, randomly oriented GNR assemblies on plain silicon wafers. Several ways to further optimize the strategy suggested are discussed.

## Background

Surface-enhanced Raman scattering (SERS) is a sensitive spectroscopic method to detect molecular vibrations on or near metallic surfaces supporting plasmonic excitation [1,2]. At present, it is generally accepted that the SERS spectra can be greatly enhanced, owing to the two mechanisms [3,4]. Specifically, the electromagnetic mechanism [3] is related to the local resonant plasmonic fields near metal nanostructures [5], whereas the so-called chemical contribution [4] is due to the formation of a charge transfer adsorption band between the Raman scattering molecules and the metallic surface (for the discussion of the well-known publication by Fleischman et al. [6] and the early history of SERS, see, e.g., [7]). The electromagnetic mechanism makes the major contribution to the SERS effect because it is both the incident and the Raman emitted field that are enhanced by the plasmonic nanostructures on the surface, thus leading to the well-known fourth-power law [2].

Since its discovery, the SERS technique has found numerous applications in chemical and biological sensing [8,9] (including single-molecule detection [10,11]), molecular and reaction dynamics [12], and biomedicine [13]. To date, the physical principles of SERS, its experimental implementation, and its applications in fundamental and applied sciences have been extensively reviewed [14-21]; the readers are referred to these reviews and the books [1,2,8].

Despite the enormous number of SERS-related publications, all the currently used SERS platforms can be placed into one of the following four broad classes determined according to the underlying fabrication method: (1) regular metal nanolithographic nanostructures [22,23], (2) metallic nanostructures obtained with the appropriate nanosized templates (‘film-over-spheres’ platforms) [24-30], (3) metal nanoparticles (NPs) assembled on plain substrates (e.g., silicon or glass) [31-34], and (4) ‘SERS tags’ that combine plasmonic NPs and specific Raman reporter organic molecules [15,21,35].

The fabricated SERS substrate should ensure several key features [33,36]: (1) high SERS enhancement and sensitivity, (2) large-scale uniformity, with the integral SERS enhancement variations over the entire substrate surface being less than 10% to 20%, (3) high stability and reproducibility between fabrication runs, and (4) low fabrication costs.

Owing to the presence of electromagnetic ‘hot spots’ near interparticle gaps, local SERS enhancements can be as high as 10^11^[36,37], but the surface-averaged enhancement is usually 3 orders of magnitude lower, about 10^8^ in the best experiments [38]. Moreover, these enhancements are unevenly distributed over wide areas. For example, Fang et al. [39] showed that the enhancement distribution could vary between 2.8 × 10^4^ and 4.1 × 10^10^, where the hot spots accounted for 0.0063% of the total number of sites examined but contributed about 24% to the average SERS intensity. This means that the major part of the recorded intensity can be due to the negligible percentage of the Raman molecules adsorbed just at these hottest sites. That is why numerous efforts were reported to develop various methods for the nanofabrication of large-scale SERS substrates possessing high and homogeneous electromagnetic enhancement [17,18].

Although multistage lithographic or patterning techniques produce the most reproducible SERS substrates, these methods are not cost-effective. Moreover, the lithographic SERS substrates can provide only a moderate enhancement as compared with some random assemblies [40]. In common practice, SERS substrates of the second type are fabricated by depositing a thin metal layer onto a self-assembled colloidal crystal. The plasmonic and SERS properties of such substrates are determined by the size of the colloidal templates used and the thickness of the deposited metal film. The film-over-spheres method allows the substrate structure to be precisely controlled, with the number of the necessary fabrication steps being minimal, which makes this technique more cost-effective. Furthermore, these substrates retain their SERS activity for months, even after their being exposed to high temperatures. For example, quite recently, Greeneltch et al. [41,42] have fabricated a new type of plasmonic SERS substrates in the form of silver or gold nanorods immobilized on silica or polystyrene microspheres covered by thin silver or gold films. This method produces radially oriented SERS-active pillars separated by small gaps. The surface plasmon resonance of such substrates was shown to be capable of being tuned from 330 to 1,840 nm by varying the microsphere diameter. For optimized substrates, the large-scale SERS enhancement was about 10^8^ under near-infrared (NIR) excitation (1,064 nm).

More recently, considerable interest has been aroused in novel nanoprobes named SERS tags [16,21] that combine plasmonic metal nanoparticles and organic Raman reporter molecules. Such SERS-active nanoprobes produce strong, characteristic Raman signals and can be used as convenient Raman labels for the indirect sensing of the target molecules by various versions of laser microscopic Raman spectrometry. In a sense, these Raman labels can be used in the same way as external chromophores, such as quantum dots or fluorescent dyes.

Perhaps the most simple and cost-effective strategy for the manufacture of SERS substrates is to fabricate self-assembled nanoparticle films (or metal islands [43,44]) on a plain supporting surface. Owing to the advances in synthesis technologies, there exist a lot of chemical protocols to fabricate metal nanoparticles differing in size, shape, structure, and composition [45-47]. In particular, plasmonic nanopowders [48,49] seem to be quite suitable for the simple and low-cost fabrication of SERS platforms based on random nanoparticle assemblies [50]. One of the obvious advantages of plasmonic nanopowders is that they retain their plasmonic properties under usual conditions; they can be easily dispersed in water to obtain sols of the desired concentration in a matter of seconds without any sonication, heating, etc. In particular, we have already utilized GNR powders to fabricate monolayer and fractal-like plasmonic films for SERS applications [33]. However, these substrates demonstrated a moderate analytical enhancement [42] averaged over the probe laser beam spot. One of the possible reasons was too small a number of the analyte molecules in the thin layers probed by the laser light.

In this work, we used gold nanorod (GNR) nanopowders [48] to prepare concentrated GNR sols that were then employed to deposit GNRs on an opal-like photonic crystal (OPC) film formed on a silicon wafer. Such GNR-OPC substrates combine the increased specific surface, owing to the multilayer nanosphere structure, and various spatial GNR configurations, including those with possible plasmonic hot spots [5,51]. We demonstrate here the existence of the optimal GNR deposition density for the maximal SERS effect, which turned out to be higher than that for the thick random GNR assemblies [33] formed directly on a plain silicon wafer.

## Methods

The gold nanorods were fabricated by the seed-mediated method, following Nikoobakht and El-Sayed [52], with minor modifications [53]. Briefly, the seed solution was obtained by mixing 10 mL of 0.1 M cetyltrimethylammonium bromide (CTAB) and 250 μL of 10 mM HAuCl_4_, followed by adding 1 mL of ice-cold 10 mM NaBH_4_. The seeds were aged for 2 h. The GNRs were obtained by mixing 900 mL of 0.1 M CTAB, 50 mL of 10 mM HAuCl_4_, 20 ml of 4 mM AgNO_3_, 10 mL of 0.1 M AsA, 10 ml of 1 M HCl, and 10 mL of the seed solution. The mixture was aged at 30°C for 48 h until an orange-red suspension was formed. We thereby obtained 1 L of a GNR sol with the longitudinal plasmon resonance at 810 to 820 nm and a total gold concentration of 85 mg/L.

The GNR sols were centrifuged twice at 16,000 × *g* for 1 h and then redispersed in water to remove the excess CTAB molecules. The pH of the GNR sols was adjusted to 9 by adding 0.2 M K_2_CO_3_, followed by the addition of methoxy(polyethylene glycol)-thiol (mPEG-SH; MW 5,000, Nektar Therapeutics, San Francisco, CA, USA) at a final concentration of 10 nM. The mixture was allowed to react overnight. The PEGylated (mPEG-SH-modified) rods were centrifuged at 16,000×*g* for 60 min and then redispersed in water to remove nonspecifically bound PEG molecules. The PEGylated GNRs were again centrifuged at 16,000×*g* for 1 h and redispersed in a small amount of water to a concentration of 5 g/L. To completely remove CTAB and unreacted PEG, the nanoparticles were dialyzed for 72 h, fresh water being added to them several times. Finally, these dialyzed, PEGylated, and concentrated GNRs were transferred to a sterile bottle, frozen in liquid nitrogen, and freeze-dried overnight under vacuum. The measured zeta potential of the as-prepared and redispersed PEGylated GNRs was about −20 mV. For details, the readers are referred to [48,49].

The absorption spectra of the GNRs were recorded with a Specord 250 BU UV-visible-NIR spectrometer (Analytik Jena, Jena, Germany). The transmission electron microscopy (TEM) images of the nanoparticles were obtained with a Libra-120 microscope (Carl Zeiss, Oberkochen, Germany). The zetapotential of the particles was measured before and after drying with a Zetasizer Nano-ZS instrument (Malvern Instruments, Malvern, UK).

The silica spheres were fabricated by the Stöber method [54] by adding the desired amount (from 0.1 to 1 mL) of 25% aqua ammonia to 10 mL of absolute ethanol and then magnetically stirring (500 rpm) the solution obtained for 5 min at room temperature. Thereafter, 0.3 mL of tetraethyl orthosilicate was added dropwise, and the suspension was stirred for 1 h and then left to stay overnight without stirring. The size of the silica spheres (200 nm in our case) is governed by the amount of ammonia added. The fabricated silica spheres were deposited by spin coating at 2,000 rpm on silicon wafers by means of a homemade centrifuge and then heat-treated [55].

The substrates were examined by scanning electron microscopy (SEM) using a JSM-6700 F instrument (JEOL, Akishima-shi, Japan), atomic force microscopy (AFM), and absorption spectroscopy with a Shimadzu UV-3600 UV–vis spectrophotometer (Shimadzu Corporation, Kyoto, Japan). The AFM images were obtained with an INTEGRA-Therma AFM microscope (NT-MDT, Moscow, Russia) operated in the semicontact and phase-contrast modes. The overall resolution was 512 × 512 points for a 2 × 2 μm^2^ region. The SERS spectra were measured with an HR800 micro-Raman spectrometer (HORIBA, Jobin Yvon, Kyoto, Japan) combined with a laser confocal microscope. To estimate the thickness of the silica film, we used the microscope of the HR800 spectrometer equipped with a ×100 objective. By comparing between the film images obtained with the microscope focused onto the inner and outer film boundaries, we found that each spin coating run formed one to three layers of silica spheres on the wafer.

To fabricate SERS substrates, we used concentrated GNR sols obtained by the redispersion of 12 mg of GNP powder in 1 mL of distilled water. A drop of a GNR sol of controllable volume was placed on a film of silica spheres on a silicon wafer and dried at room temperature. This process was repeated several times to attain the desired surface and volume densities of the GNRs embedded in and deposited on the OPC film. For comparative purposes, we also fabricated SERS substrates by depositing GNR sols differing in concentration directly on plain silicon wafers as described previously in [33].

## Results and discussion

### Properties of GNR powders

**Figure 1:**
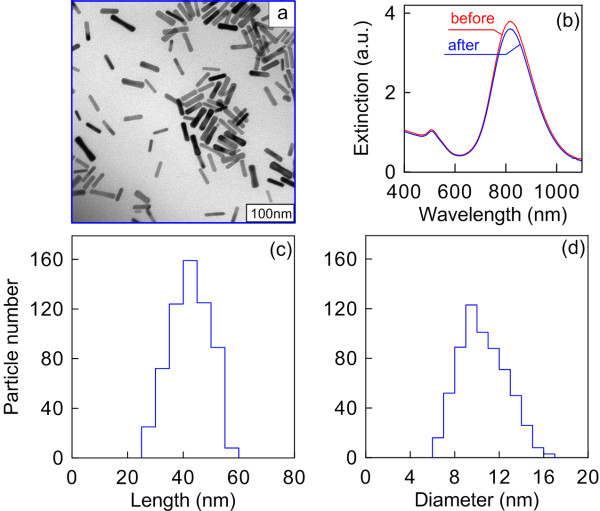
**TEM image, extinction spectra, GNR length distribution histogram, and GNR diameter distribution histogram.** (**a**) TEM image of a GNR powder redispersed in water. (**b**) Extinction spectra of the as-prepared GNRs and GNR powder after freeze-drying. (**c**) GNR length distribution histogram. (**d**) GNR diameter distribution histogram. The average length and diameter of GNRs are both in nanometers.

Figure 1a shows a TEM image of a GNP nanopowder redispersed in water. The size and shape of the nanoparticles practically do not differ from those the as-prepared GNRs had before freeze-drying. Accordingly, there are no essential differences between the extinction spectra of the samples recorded prior to and after freeze-drying (Figure 1b). Both spectra reveal a typical longitudinal plasmon resonance near 820 nm and a minor transverse resonance maximum around 510 nm. The high ratio between the longitudinal and transverse resonance amplitudes points to the high quality of the samples and to the minor presence of by-product particles therein [56].

The powdered GNR particles have a typical cigar-like shape; their length and diameter distributions are shown in Figure 1c,d, respectively. According to the results of reckoning for 600 particles, they are 44.8 ± 7.6 nm in length and 11.2 ± 2.3 nm in diameter. Their distinctive feature is high solubility at high concentrations (up to 50 mg/mL), hundreds of times as high as the typical concentrations attainable with seed-mediated synthesis [52,53,57].

### Formation and characterization of silica films

**Figure 2:**
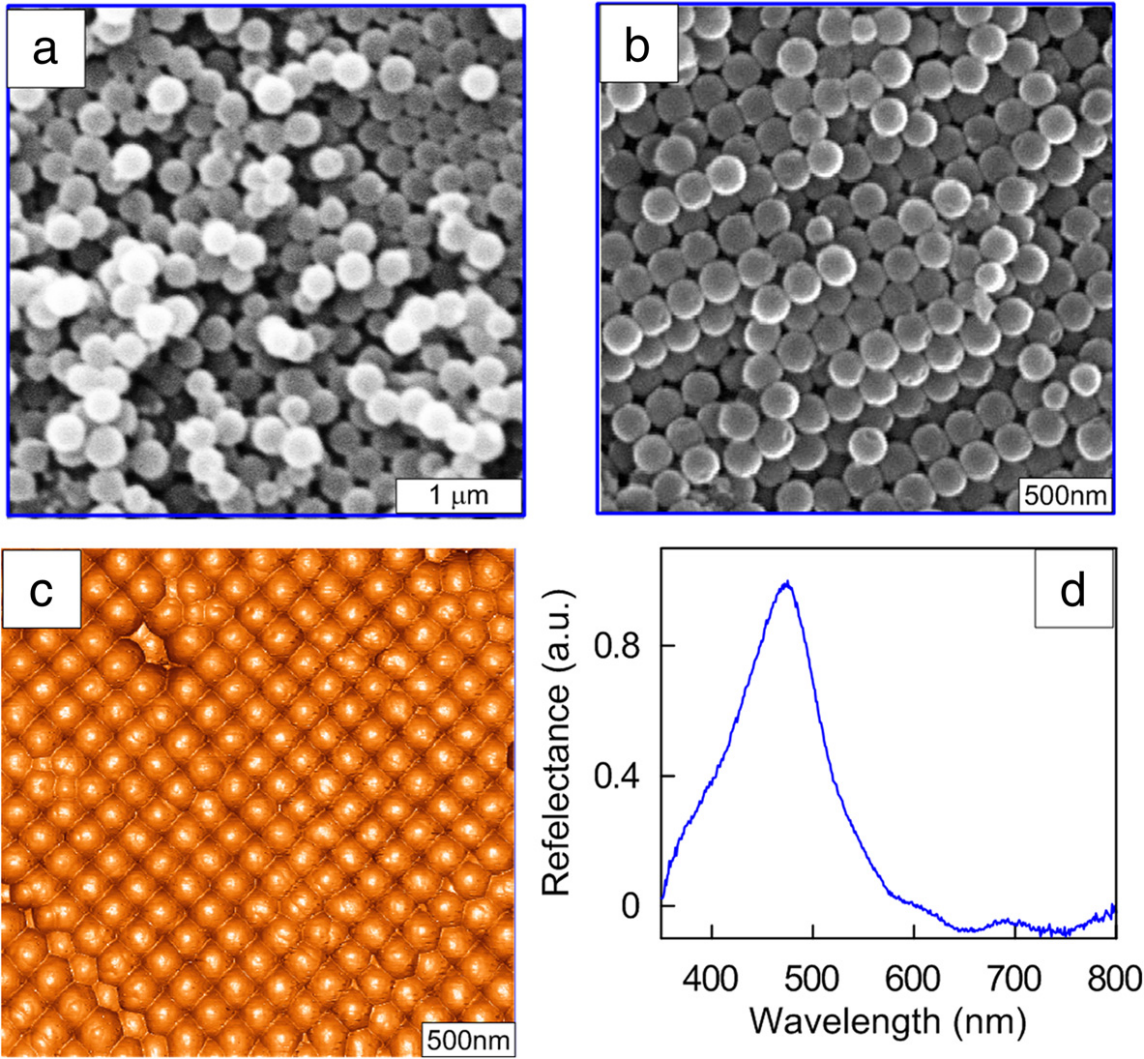
**SEM and AFM images of opal-like photonic crystals and the bandgap zone.** Respective SEM (**a**, **b**) and AFM (**c**) images of thin (**a**) and thick (**b**, **c**) opal-like photonic crystals formed by depositing 200-nm silica spheres by spin coating on a silicon substrate. (**d**) The bandgap zone centered around 500 nm as revealed from the reflectance spectrum.

According to the data of [58], the typical size polydispersity of the Stöber spheres (100 to 200 nm in diameter), as determined in terms of the full width at half maximum Δ*d*/*d*_max_, is about 20% (see, e.g., panels c and d in Figure three in [58]). Because of the surface defects, the first spin-coated layers were inhomogeneous, with some ordered islands present. After 5 to 10 spin coating cycles, there formed more ordered structures similar to the opal-like photonic crystals [59,60] (Figure 2a,b,c).

As the number of the spin-coated layers of silica spheres was increased, there formed ordered structures characterized by a typical photonic bandgap appearing in their reflectance spectrum (Figure 2d). Because of the intrinsic polydispersity of the Stöber silica spheres and packing defects, the photonic bandgap width in Figure 2d is significantly greater than that for true high-contrast photonic crystals [61]. Nevertheless, even a partial orderliness in thick opal-like films gives a characteristic spectrum with a bandgap near 500 nm. Increasing the film thickness augmented the contribution from SiO_2_ to the SERS spectra recorded.

### GNR-Si and GNR-OPC substrates

For comparative measurements, we used densely packed and fractal-like GNR films deposited on silicon wafers. The structure of such substrates is shown in Additional file 1: Figure S1. In particular, the fractal-like GNR assemblies were shown to have a maximum SERS enhancement in comparison with single-particle and densely packed monolayers [33]. For dilute GNR sols, the GNR assemblies demonstrated an island structure after deposition on a silicon wafer and drying in air (see, for example, Additional file 1: Figure S2). It should be emphasized that the plasmonic properties of single GNRs and GNR assemblies differ substantially because of the strong electromagnetic coupling between neighboring particles [62] (Additional file 1: Figure S3). It follows from Additional file 1: Figure S3 that the interaction of particles in dense films leads to the broadening and red shifting of the principal longitudinal dipole resonance and reduction of its magnitude. What is more, there emerge minor resonances due to the higher (nondipole) modes of plasmonic excitations. The abovementioned sudden change in the plasmon spectra of films formed from nanorods is a negative factor from the standpoint of SERS applications. Note for comparison that the more complex techniques of application of metal films over 2-D colloidal silica or polystyrene crystals provide for a controllable plasmonic shift towards the near-IR region without any serious impairment of the spectral quality.

**Figure 3:**
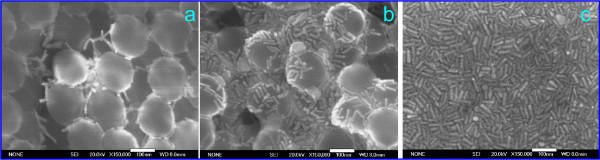
**SEM images of mesoporous silica films differing in GNR deposition density.** (**a**) Low. (**b**) Medium. (**c**) High. Note that the densely packed GNR layer (right-hand image) is similar to the fractal-like GNR assembly on a silicon wafer (Additional file 1: Figure S2b). The white bars are 100 nm long.

**Figure 4:**
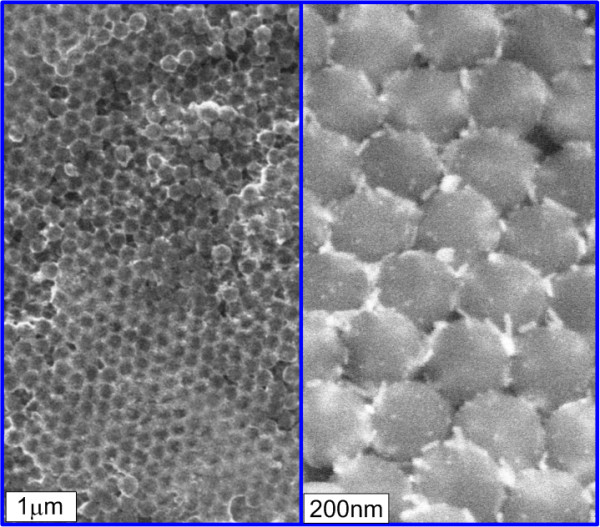
**SEM images of a GNR-OPC substrate at a low (left) and a high (right) resolution.** The light regions near silica spheres (left image) correspond to the deposited GNRs that are clearly seen in the enlarged image (right).

To obtain GNR-OPC substrates, we prepared nanorod sols with a GNR powder concentration of 12 mg/mL in water. This concentration approximately corresponded to the maximum enhancement of the SERS spectra of rhodamine 6G and 4-aminthiophenol (see Additional file 1: Figure S4). During the course of deposition, the GNRs gradually filled up the interstitial space. While the amount of the deposited particles was small, they completely entered into pores, with only solitary particles remaining on the surface (Figure 3a). Thereafter, islands of gold nanorods formed on the film surface that overlapped at the points of contact between silica spheres (Figure 3b). Finally, when the amount of the deposited GNRs became large enough, we observed some kind of plain GNR film without any fingerprints of silica spheres (Figure 3c). Note that we purposefully selected in Figure 3 an irregular area of silica spheres with large pores in order to illustrate the process of the pores being filled up with gold nanorods. Additional information is presented in Figure 4 for an area having a colloidal crystal structure.

### SERS spectra recorded with GNR-Si and GNR-OPC substrates

To compare between the analytical enhancements [42] provided by the SERS structures formed, we used the rhodamine 6G (R6G) dye as a test analyte. In a typical SERS measurement protocol, 2.5 μL of an R6G solution in ethanol 80 μM in concentration was applied onto the surface of the substrate under study. The average surface area occupied by the dye droplet spread on the substrate was around 7 mm^2^. Measurements were mainly taken using radiation from a He-Ne laser (wavelength 632.8 nm, power in the beam spot approximately 5 mW). The laser beam spot diameter was around 20 μm, and the signal accumulation time came to 10 s (the signal was averaged over 10 measurements). With the test conditions remaining the same, SERS signals were measured from the R6G dye applied onto GNR-Si and GNR-OPC substrates differing in thickness of the opal-like film.

**Figure 5:**
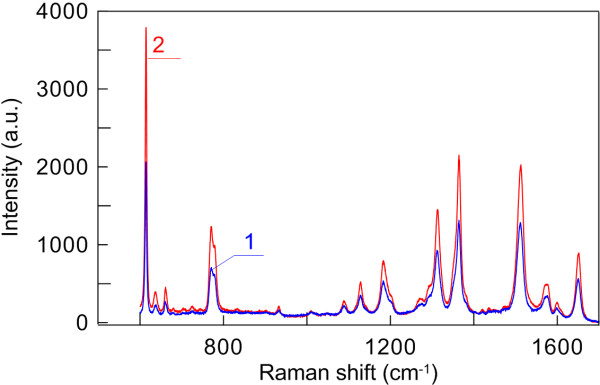
**SERS spectra of 80 μM rhodamine 6G solution applied onto GNR-Si (1) and thin GNR-OPC (2) substrates.** Excited at 632.8 nm.

Figure 5 shows the SERS spectra of the 80 μM rhodamine 6G solution applied onto a GNR-Si (spectrum 1) and a GNR-OPC (spectrum 2) substrate excited at 632.8 nm. Evidently, the integral analytical enhancement [42] of the GNR-OPC substrate is from two to five times as high as that of the simple fractal-like GNR assembly on silicon. A common property of SERS measurements is that the integral enhancement depends on the particular Raman line selected for the purpose. The fundamental SERS enhancement [41,42] is determined by several important factors that are difficult to take into account for mesoporous substrates. For a detailed discussion of this point, the readers are referred to the comprehensive analysis by Le Ru et al. [36].

**Figure 6:**
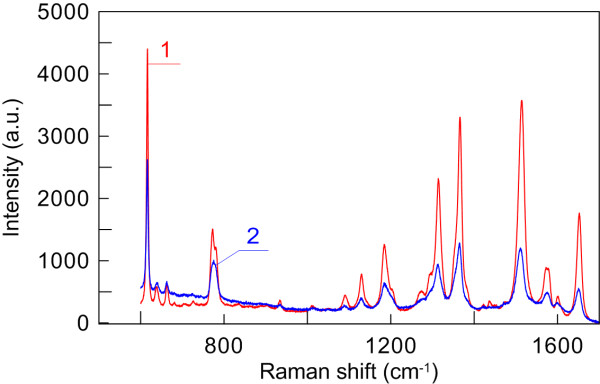
**SERS spectra of 80 μM rhodamine 6G solution applied onto thin (1) and thick (2) GNR-OPC substrates.** Excited at 632.8 nm.

In Figure 6, we compare between the SERS spectra of the 80 μM rhodamine 6G solution applied onto ‘thin’ and ‘thick’ GNR-OPC substrates. This classification roughly corresponds to the number of the deposited silica layers, which is less than 10 in the former case and more than 10 in the latter. However, in both cases, the pores between silica spheres are densely covered by GNRs, but GNRs fail to cover the silica spheres completely. Surprisingly enough, the maximum SERS enhancement is observed with thin rather than thick substrates (*cf.* spectra 1 and 2 in Figure 6). It should be noted that the elevated tail in SERS spectrum 2 is due exactly to a thick silica film contribution. For thin substrates, the baseline is flat (similar to that for spectrum 1 in Figure 6). Moreover, for extremely thick substrates (about 1 to 2 mm thick), the SERS enhancement falls down, and we observe a monotonous contribution from the underlying silica opal (data not shown).

Taking into account the analytical SERS enhancement coefficient of GNR-Si substrates [33] (2.5 × 10^3^), we estimate the analytical enhancement coefficient of GNR-OPC substrates to be on the order of 10^4^. We suppose that the additional SERS enhancement in the GNR-OPC substrates is due to several factors. First, such substrates have an increased specific surface and, accordingly, more Raman molecules capable of contributing to the measured spectra. Second, the highly inhomogeneous arrangement of GNRs in thin GNR-OPC films can produce more electromagnetic hot spots. Finally, additional contribution can come from multiple scattering within thick opal films, though this assumption needs to be specially studied.

## Conclusions

In this work, we have studied a very simple technique to fabricate SERS substrates using wet chemical approaches only. Our approach is based on the use of a plasmonic powder of gold nanorods that are applied in a concentrated form onto an opal-like substrate of silicon nanospheres. As compared with the previously studied randomly oriented mono- and polylayers of gold nanorods on a plain silicon substrate, the structures obtained by us provide for a two- to fivefold enhancement of the SERS signal. The main mechanisms behind this effect are apparently the increase of the number of reporter molecules adsorbed on the mesoporous substrate and the increase of the number of electromagnetic hot spots. Of course, the analytical SERS enhancement coefficients attained with our structures are a few orders of magnitude lower than those for such structures as the silver-immobilized nanorod assembly [41,42]. However, the principal advantage of our approach is its exceptional simplicity, for it requires no special procedures of vacuum deposition on colloidal crystals.

Several ways are possible to optimize the method described here. First, it seems advisable to replace gold nanorods with silver-coated nanorods [63] or to investigate other types of nonspherical gold or silver nanoparticles [64]. For example, Zhang et al. [65] fabricated SERS substrates based on large-scale metallic thin films assembled from size-selected silver nanoplates with tunable plasmonic properties. It was shown [65] that the aggregation of silver nanoplates with sharp corners produces hot spots between the corner gaps, thus leading to strong electromagnetic SERS enhancement. Unfortunately, unlike that of gold nanorods, the high-yield fabrication of monodisperse silver nanorods is not an easy task [66,67], and a recent review of this issue has been published by Negri and Dluhy [68]. However, gold nanorods can be used as convenient templates for subsequent silver coating to ensure flexible tuning of the localized plasmon resonance from near-infrared (e.g., 900 nm) to visible (e.g., 580 nm) [69]. Our preliminary results show that the Au@Ag core-shell nanorod assemblies demonstrate better SERS performance as compared to aggregated gold nanorod films. Our full 3-D finite-difference time-domain simulations [70] confirm the existence of enhanced local electromagnetic hot spots that are more intensive in the case of random assemblies of silver-coated nanorods. Investigations along these lines are under way at our laboratories, and the results will be published elsewhere.

## Abbreviations

AFM: Atomic force microscopy; CTAB: Cetyltrimethylammonium bromide; GNRs: Gold nanorods; GNR-OPC: Gold nanorods on a mesoporous colloidal silica film; GNR-Si: Gold nanorods on a silicon substrate; OPC: Mesoporous colloidal silica film; R6G: Rhodamine 6G; SERS: Surface-enhanced Raman (scattering) spectroscopy; T(S) EM: Transmission (scanning) electron microscopy.

## Competing interests

The authors declare that they have no competing interests.

## Authors' contributions

MYuT, BNK, VAK, and PST searched for the sample processing regimens, SEM, TEM, AFM, spectroscopic, and SERS measurements. MIS provided the opal-like substrates. VNB coordinated the project as a whole. MYuT provided a preliminary version of the manuscript. NGK analyzed all data, wrote the final version of the manuscript, and arranged all figures. All authors read and approved the final manuscript.

## Supplementary Material

Additional file 1 **Supporting information.** The file contains Figures S1 to S4. Click here for file

## References

[B1] Aroca R Surface-Enhanced Vibrational Spectroscopy 2006 Chichester: Wiley

[B2] Le R EC, Etchegoin PG: Principles of Surface Enhanced Raman Spectroscopy 2009 Amsterdam: Elsevier

[B3] Jeanmarie DL Van Duyne RP Surface Raman spectroelectrochemistry, part 1: heterocyclic, aromatic, and aliphatic amines adsorbed on the anodized silver electrode J Electroanal Chem 1977 8 120

[B4] Otto A The ‘chemical’ (electronic) contribution to surface-enhanced Raman scattering J Raman Spectrosc 2005 8 497 509 10.1002/jrs.1355

[B5] Khlebtsov NG T-matrix method in plasmonics J Quant Spectr Radiat Transfer 2013 8 184 217

[B6] Fleischmann M Hendra PJ McQuillan AJ Raman spectra of pyridine adsorbed at a silver electrode Chem Phys Lett 1974 8 163 166 10.1016/0009-2614(74)85388-1

[B7] Haynes CL Yonzon CR Zhang X Van Duyne R Surface-enhanced Raman sensors: early history and the development of sensors for quantitative biowarfare agent and glucose detection J Raman Spectrosc 2005 8 471 484 10.1002/jrs.1376

[B8] Anker JN Hall WP Lyandres O Shan NC Zhao J Van Duyne RP Biosensing with plasmonic nanosensors Nature Material 2008 8 442 453 10.1038/nmat2162 18497851

[B9] Schlücker S Surface Enhanced Raman Spectroscopy. Analytical, Biophysical and Life Science Applications 2011 Chichester: Wiley

[B10] Kneipp K Wang Y Kneipp H Perelman LT Itzkan I Dasari RR Feld MS Single molecule detection using surface-enhanced Raman scattering (SERS) Phys Rev Lett 1997 8 1667 1670 10.1103/PhysRevLett.78.1667

[B11] Nie S Emory SR Probing single molecules and single nanoparticles by surface-enhanced Raman scattering Science 1997 8 1102 1106 10.1126/science.275.5303.1102 9027306

[B12] Lai SCS Koper MTM Ethanol electro-oxidation on platinum in alkaline media Phys Chem Chem Phys 2009 8 10446 10456 10.1039/b913170a 19890531

[B13] Khlebtsov NG Dykman LA Optical properties and biomedical applications of plasmonic nanoparticles J Quant Spectr Radiat Transfer 2010 8 1 35 10.1016/j.jqsrt.2009.07.012

[B14] Ko H Singamaneni S Tsukruk VV Nanostructured surfaces and assemblies as SERS media Small 2008 8 1576 1599 10.1002/smll.200800337 18844309

[B15] Qian X-M Nie SM Single-molecule and single-nanoparticle SERS: from fundamental mechanisms to biomedical applications Chem Soc Rev 2008 8 912 920 10.1039/b708839f 18443676

[B16] Álvarez-Puebla RA Liz-Marzán LM Traps and cages for universal SERS detection Chem Soc Rev 2012 8 43 51 10.1039/c1cs15155j 21818469

[B17] Lin X-M Cui Y Xu Y-H Ren B Tian Z-Q Surface-enhanced Raman spectroscopy: substrate-related issues Anal Bioanal Chem 2009 8 1729 1745 10.1007/s00216-009-2761-5 19381618

[B18] Fan MK Andrade GFS Brolo AG A review on the fabrication of substrates for surface enhanced Raman spectroscopy and their applications in analytical chemistry Anal Chim Acta 2011 8 7 25 10.1016/j.aca.2011.03.002 21504806

[B19] Cialla D März A Böhme R Theil F Weber K Schmitt M Popp J Surface-enhanced Raman spectroscopy (SERS): progress and trends Anal Bioanal Chem 2012 8 27 54 10.1007/s00216-011-5631-x 22205182

[B20] Tong L Zhu T Li Z Approaching the electromagnetic mechanism of surface-enhanced Raman scattering: from self-assembled arrays to individual gold nanoparticles Chem Soc Rev 2011 8 1296 1304 10.1039/c001054p 21125088

[B21] Wang Y Yan B Chen L SERS tags: novel optical nanoprobes for bioanalysis Chem Rev 2013 8 1391 1428 10.1021/cr300120g 23273312

[B22] Haynes CL Van Duyne RP Nanosphere lithography: a versatile nanofabrication tool for studies of size-dependent nanoparticle optics J Phys Chem B 2001 8 5599 5611 10.1021/jp010657m

[B23] Kosuda KM Bingham JM Wustholz KL Van Duyne RP Nanostructures and surface-enhanced Raman spectroscopy Compr Nanosci Technol 2011 8 263 301

[B24] Baia M Baia L Astilean S Gold nanostructured films deposited on polystyrene colloidal crystal templates for surface-enhanced Raman spectroscopy Chem Phys Lett 2005 8 3 8 10.1016/j.cplett.2005.01.052

[B25] Lu L Randjelovic I Capek R Gaponik N Yang J Zhang H Eychmüller A Controlled fabrication of gold-coated 3D ordered colloidal crystal films and their application in surface-enhanced Raman spectroscopy Chem Mater 2005 8 5731 5736 10.1021/cm051473d

[B26] Mahajan S Abdelsalam M Suguwara Y Cintra S Russell A Baumberg J Bartlett P Tuning plasmons on nano-structured substrates for NIR-SERS Phys Chem Chem Phys 2007 8 104 109 10.1039/b611803h 17164891

[B27] Liu X Sun C-H Linn NC Jiang B Jiang P Wafer-scale surface-enhanced Raman scattering substrates with highly reproducible enhancement J Phys Chem C 2009 8 14804 14811 10.1021/jp905065z

[B28] Liu X Sun C-H Linn NC Jiang B Jiang P Templated fabrication of metal half-shells for surface-enhanced Raman scattering Phys Chem Chem Phys 2010 8 1379 1387 10.1039/b919916k 20119616

[B29] Rao Y Tao Q An M Rong C Dong J Dai Y Qian W Novel and simple route to fabricate 2D ordered gold nanobowl arrays based on 3D colloidal crystals Langmuir 2011 8 13308 13313 10.1021/la203158q 21932785

[B30] Liu G Li Y Duan G Wang J Liang C Cai W Tunable surface plasmon resonance and strong SERS performances of Au opening-nanoshell ordered arrays ACS Appl Mater Interfaces 2012 8 1 5 10.1021/am201455x 22171761

[B31] Nikoobakht B El-Sayed MA Surface-enhanced Raman scattering studies on aggregated gold nanorods J Phys Chem A 2003 8 3372 3378 10.1021/jp026770+

[B32] Kuncicky DM Prevo BG Velev OD Controlled assembly of SERS substrates templated by colloidal crystal films J Mater Chem 2006 8 1207 1211 10.1039/b512734c

[B33] Khlebtsov BN Khanadeev VA Panfilova EV Minaeva SA Tsvetkov MY Bagratashvili VN Khlebtsov NG Surface-enhanced Raman scattering platforms on the basis of assembled gold nanorods Nanotechnologies in Russia 2012 8 359 369 10.1134/S1995078012040064

[B34] Farcau C Potara M Leordean C Boca S Astilean S Reliable plasmonic substrates for bioanalytical SERS applications easily prepared by convective assembly of gold nanocolloids Analyst 2013 8 546 552 10.1039/c2an36440a 23171872

[B35] Gabudean AM Focsan M Astilean S Gold nanorods performing as dual-modal nanoprobes via metal-enhanced fluorescence (MEF) and surface-enhanced Raman scattering (SERS) J Phys Chem C 2012 8 12240 12249 10.1021/jp211954m

[B36] Le Ru EC Blackie E Meyer M Etchegoin PG Surface enhanced Raman scattering enhancement factors: a comprehensive study J Phys Chem C 2007 8 13794 13803 10.1021/jp0687908

[B37] Blaber MG Schatz GC Extending SERS into the infrared with gold nanosphere dimers Chem Commun 2011 8 3769 3771 10.1039/c0cc05089j 21340050

[B38] Wustholz KL Henry AI McMahon JM Freeman RG Valley N Piotti ME Natan MJ Schatz GC Van Duyne RP Structure-activity relationships in gold nanoparticle dimers and trimers for surface-enhanced Raman spectroscopy J Am Chem Soc 2010 8 10903 10910 10.1021/ja104174m 20681724

[B39] Fang Y Seong NH Dlott DD Measurement of the distribution of site enhancements in surface-enhanced Raman scattering Science 2008 8 388 392 10.1126/science.1159499 18583578

[B40] Natan MJ Concluding remarks. Surface enhanced Raman scattering Faraday Discuss 2006 8 321 328 16833126 10.1039/b601494c

[B41] Greeneltch NG Blaber MG Schatz GC Van Duyne RP Plasmon-sampled surface-enhanced Raman excitation spectroscopy on silver immobilized nanorod assemblies and optimization for near infrared (λ_ex_ = 1064 nm) studies J Phys Chem C 2013 8 2554 2558 10.1021/jp310846j

[B42] Greeneltch NG Blaber MG Henry A-I Schatz GC Van Duyne RP Immobilized nanorod assemblies: fabrication and understanding of large area surface-enhanced Raman spectroscopy substrates Anal Chem 2013 8 2297 2303 10.1021/ac303269w 23343409

[B43] Zhurikhina VV Brunkov PN Melehin VG Kaplas T Svirko Y Rutckaia VV Lipovskii AA Self-assembled silver nanoislands formed on glass surface via out-diffusion for multiple usages in SERS applications Nanoscale Res Lett 2012 8 676 10.1186/1556-276X-7-676 23244007 PMC3563545

[B44] Zhu SQ Zhang T Guo XL Wang QL Liu X Zhang XY Gold nanoparticle thin films fabricated by electrophoretic deposition method for highly sensitive SERS application Nanoscale Res Lett 2012 8 613 10.1186/1556-276X-7-613 23130848 PMC3502474

[B45] Dykman L Khlebtsov N Gold nanoparticles in biomedical applications: recent advances and perspectives Chem Soc Rev 2012 8 2256 2282 10.1039/c1cs15166e 22130549

[B46] Zhao P Li N Astruc D State of the art in gold nanoparticle synthesis Coord Chem Rev 2013 8 638 665 10.1016/j.ccr.2012.09.002

[B47] Tan KS Cheong KY Advances of Ag, Cu, and Ag-Cu alloy nanoparticles synthesized via chemical reduction route J Nanopart Res 2013 8 1 29

[B48] Khlebtsov BN Panfilova EV Terentyuk GS Maksimova IL Ivanov AV Khlebtsov NG Plasmonic nanopowders for photothermal therapy of tumors Langmuir 2012 8 8994 9002 10.1021/la300022k 22404289

[B49] Khlebtsov BN Khanadeev VA Panfilova EV Pylaev TE Bibikova OA Staroverov SA Bogatyrev VA Dykman LA Khlebtsov NG New types of nanomaterials: powders of gold nanospheres, nanorods, nanostars, and gold–silver nanocages Nanotechnologies in Russia 2013 8 209 219 10.1134/S1995078013020092

[B50] Tsvetkov MY Khlebtsov BN Panfilova EV Bafratashvili VN Khlebtsov NG Gold nanorods as a promising technological platform for SERS-analytics Russian Chem J 2012 8 83 90 (in Russian)

[B51] Stockman MI Nanoplasmonics: past, present, and glimpse into future Opt Express 2011 8 22029 22106 10.1364/OE.19.022029 22109053

[B52] Nikoobakht B El-Sayed MA Preparation and growth mechanism of gold nanorods (NRs) using seed-mediated growth method Chem Mater 2003 8 1957 1962 10.1021/cm020732l

[B53] Khlebtsov B Khanadeev V Khlebtsov N A new T-matrix solvable model for nanorods: TEM-based ensemble simulations supported by experiments J Phys Chem C 2011 8 6317 6323

[B54] Stöber W Fink A Bohn E Controlled growth of monodisperse silica spheres in the micron size range J Colloid Interface Sci 1968 8 62 69 10.1016/0021-9797(68)90272-5

[B55] Tsvetkov MY Bagratashvili VN Panchenko VY Rybaltovskii AO Samoylovich MI Timofeev MA Plasmon resonances of silver nanoparticles in silica based mesostructured films Nanotechnologies in Russia 2011 8 619 624 10.1134/S1995078011050156

[B56] Khlebtsov BN Khanadeev VA Khlebtsov NG Observation of extra-high depolarized light scattering spectra from gold nanorods J Phys Chem C 2008 8 12760 12768 10.1021/jp802874x 24522336

[B57] Ratto F Matteini P Rossi F Pini R Size and shape control in the overgrowth of gold nanorods J Nanopart Res 2010 8 2029 2036 10.1007/s11051-009-9712-0

[B58] Khlebtsov BN Khanadeev VA Khlebtsov NG Determination of the size, concentration, and refractive index of silica nanoparticles from turbidity spectra Langmuir 2008 8 107 110 10.1021/la8010053 18590302

[B59] Busch K John S Photonic band gap formation in certain self-organizing systems Phys Rev E 1998 8 3896 3908

[B60] Lopez C Materials aspects of photonic crystals Adv Mater 2003 8 1679 1704 10.1002/adma.200300386

[B61] Bertone JF Jiang P Hwang KS Mittleman DM Colvin VL Thickness dependence of the optical properties of ordered silica-air and air-polymer photonic crystals Phys Rev Lett 1999 8 300 303 10.1103/PhysRevLett.83.300

[B62] Jain PK El-Sayed MA Plasmonic coupling in noble metal nanostructures Chem Phys Lett 2010 8 153 164 10.1016/j.cplett.2010.01.062

[B63] Zong S Wang Z Yang J Wang C Xu S Cui Y A SERS and fluorescence dual mode cancer cell targeting probe based on silica coated Au@Ag core–shell nanorods Talanta 2012 8 368 375 22841094 10.1016/j.talanta.2012.04.047

[B64] Wang C Chen Y Ma Z Wang T Su Z Generalized fabrication of surfactant-stabilized anisotropic metal nanoparticles to amino-functionalized surfaces: application to surface-enhanced Raman spectroscopy J Nanosci Nanotechno 2008 8 5887 5895 10.1166/jnn.2008.222 19198322

[B65] Zhang X-Y Hu A Zhang T Lei W Xue X-J Zhou Y Duley WW Self-assembly of large-scale and ultrathin silver nanoplate films with tunable plasmon resonance properties ACS Nano 2011 8 9082 9092 10.1021/nn203336m 21955107

[B66] Pietrobon B McEachran M Kitaev V Synthesis of size-controlled faceted pentagonal silver nanorods with tunable plasmonic properties and self-assembly of these nanorods ACS Nano 2009 8 21 26 10.1021/nn800591y 19206244

[B67] Mahmoud MA El-Sayed MA Different plasmon sensing behavior of silver and gold nanorods J Phys Chem Lett 2013 8 1541 1545 10.1021/jz4005015 26282312

[B68] Negri P Dluhy RA Ag nanorod based surface-enhanced Raman spectroscopy applied to bioanalytical sensing J Biophotonics 2013 8 20 35 10.1002/jbio.201200133 23175392 PMC3767285

[B69] Khlebtsov B Khanadeev V Khlebtsov N Tunable depolarized light scattering from gold and gold/silver nanorods Phys Chem Chem Phys 2010 8 3210 3218 10.1039/b925102b 20237711

[B70] Taflove A Computational Electrodynamics: The Finite-Difference Time-Domain Method 1995 Boston: Artech House

