# Supplementary material for: SERS substrates formed by gold nanorods deposited on colloidal silica films
Source: Nanoscale Res Lett. 2013 May 22;8(1):250. doi: 10.1186/1556-276X-8-250 (PMC3664605; doi:10.1186/1556-276X-8-250)
Supplement: Additional file 1 — Supporting information. The file contains Figures S1 to S4. [file 1556-276X-8-250-S1.doc]

**Supporting Information**

**SERS substrates formed by gold nanorods deposited on colloidal silica films**

Mikhail Yu. Tsvetkov^1,#^, Boris N. Khlebtsov^2,#^, Vitaly A. Khanadeev^2^, Victor N. Bagratashvili^1^,
Peter S. Timashev^1^, Mikhail I. Samoylovich^3^, Nikolai G. Khlebtsov^2,4,*^

^1^Institute of Laser and Information Technologies, Russian Academy of Sciences, Advanced Laser Technologies Division, Moscow, 2 Pionerskaya Ulitsa, Troitsk, 142190, Russia

^2^Institute of Biochemistry and Physiology of Plants and Microorganisms,
Russian Academy of Sciences, 13 Prospekt Entuziastov, Saratov 410049, Russia

^3^ Central Research Technological Institute “TECHNOMASH”,
4, I. Franko Ulitsa, Moscow 121108, Russia

^4^ Saratov State University, 83 Astrakhanskaya Ulitsa, Saratov 410012, Russia

* Correspondence: [khlebtsov@ibppm.sgu.ru](mailto:khlebtsov@ibppm.sgu.ru)

^#^ Equal contribution


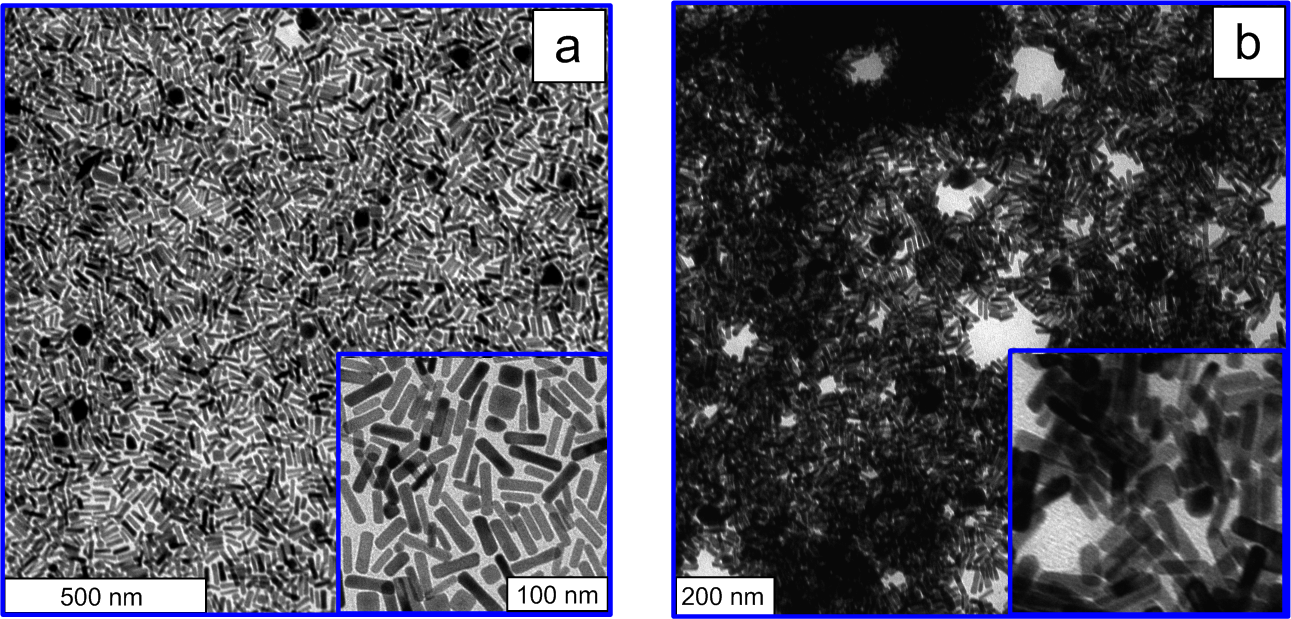


Figure S1 TEM images of a monolayer densely-packed (a) and fractal-like (b) films of GNRs deposited on a silicon wafer.


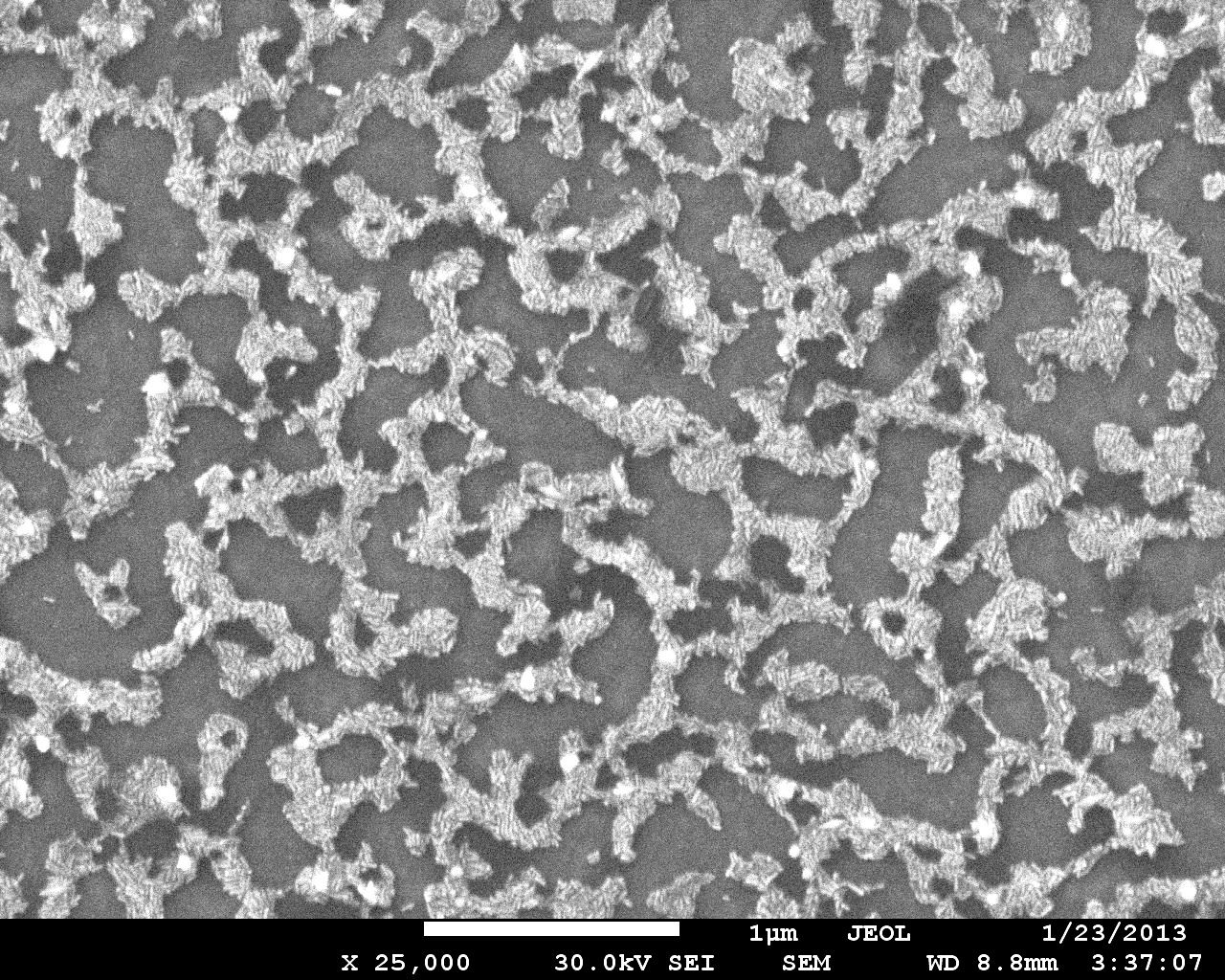


Figure S2 Island-like structure of GNRs after deposition on a silicon wafer and drying on air.


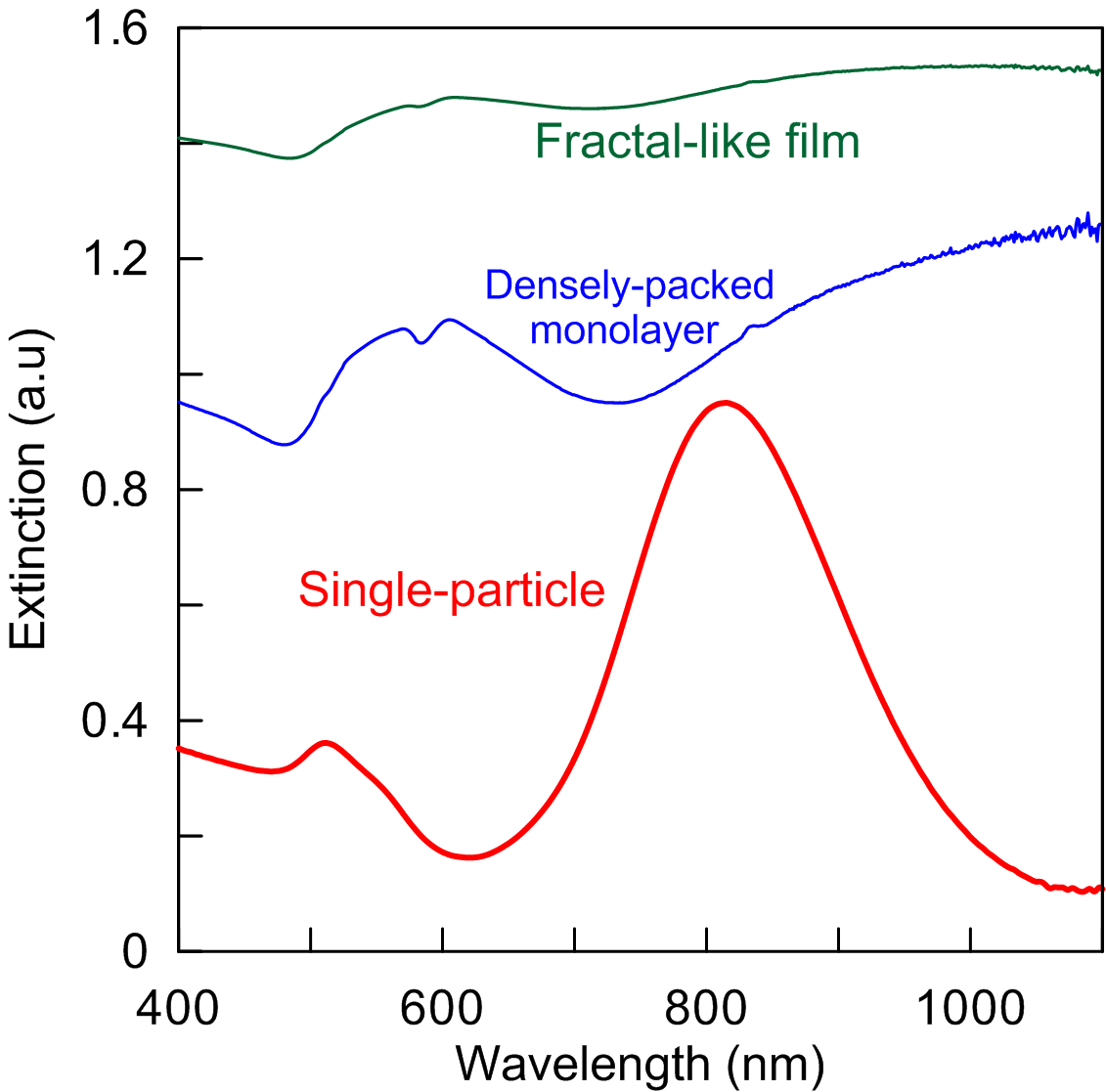


Figure S3 Extinction spectra of diluted suspension (red single-particle curve), densely-packed monolayer (see Figure S1a), and fractal-like thick film (see Figure S1b).


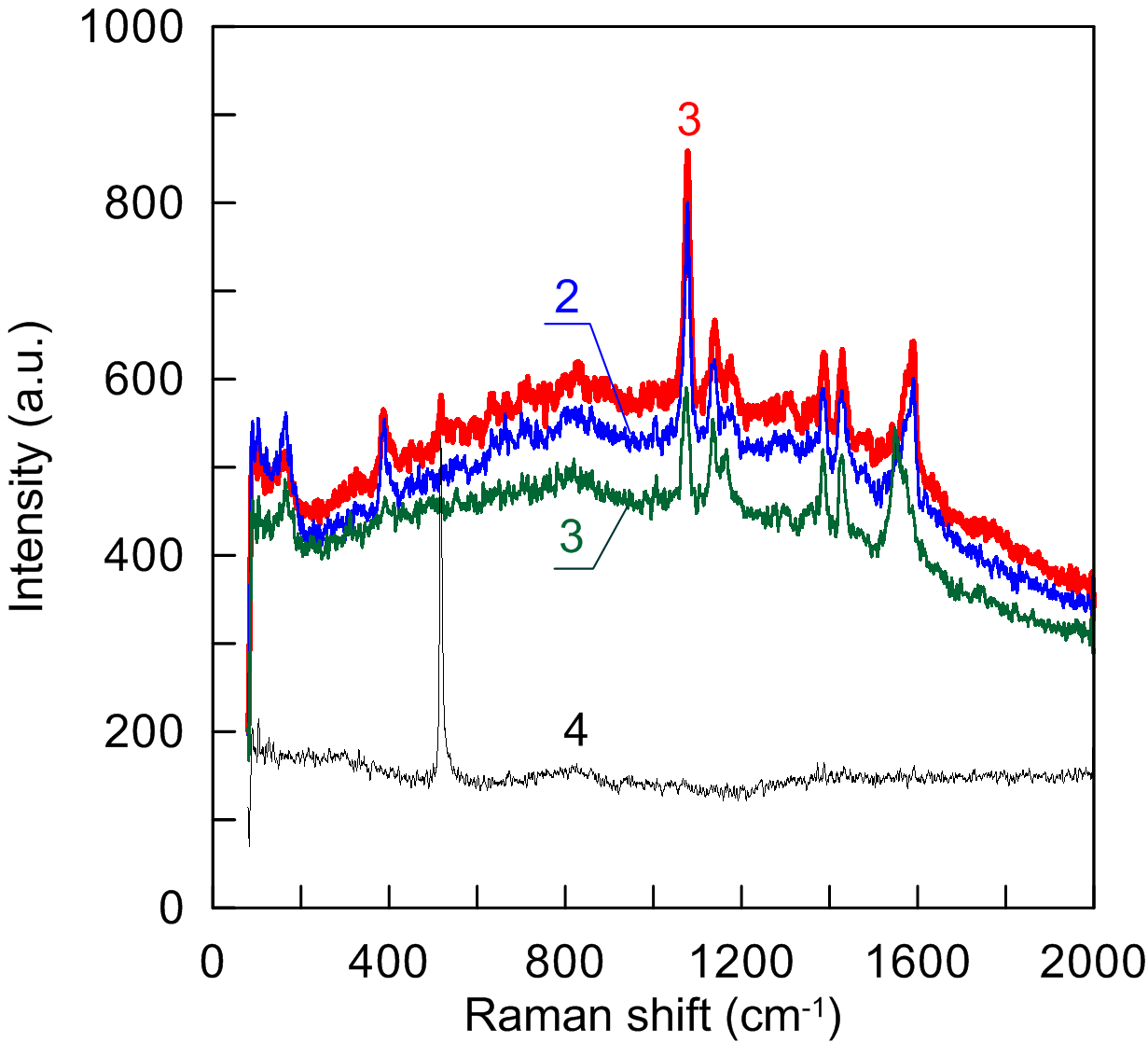


Figure S4 SERS spectra of the 400 μM 4-aminothiophenol recorded with a fractal-like GNR substrate for concentration of GNRs in solution 48 (1), 24 (2), 12 (3), 6 (4) mg/mL. Laser excitation at 633 nm.
